# The IgH 3’ regulatory region and c-myc-induced B-cell lymphomagenesis

**DOI:** 10.18632/oncotarget.12535

**Published:** 2016-10-08

**Authors:** Nour Ghazzaui, Alexis Saintamand, Hussein Issaoui, Christelle Vincent-Fabert, Yves Denizot

**Affiliations:** ^1^ Université de Limoges, Centre National de la Recherche Scientifique, CNRS UMR, France

**Keywords:** c-myc, 3 regulatory region, lymphomagenesis, transgenic mice, HDAC

## Abstract

Deregulation and mutations of c-myc have been reported in multiple mature B-cell malignancies such as Burkitt lymphoma, myeloma and plasma cell lymphoma. After translocation into the immunoglobulin heavy chain (IgH) locus, c-myc is constitutively expressed under the control of active IgH *cis*-regulatory enhancers. Those located in the IgH 3 regulatory region (3RR) are master control elements of transcription. Over the past decade numerous convincing demonstrations of 3RRs contribution to mature c-myc-induced lymphomagenesis have been made using transgenic models with various types of IgH-c-myc translocations and transgenes. This review highlights how IgH 3RR physiological functions play a critical role in c-myc deregulation during lymphomagenesis.

## INTRODUCTION

The immunoglobulin heavy chain (IgH) locus undergoes numerous changes during B-cell differentiation, affecting transcription, V(D)J accessibility to recombination, class switch recombination (CSR) and somatic hypermutation (SHM) [1, 2]. Ongoing recombination and mutation throughout B-cell development, *via* Rag1/Rag2 and AID targeting [1, 2], make the IgH locus a hotspot for translocations. Several human mature B-cell lymphomas are marked by oncogene translocations into the IgH locus [3]. Bcl-2 and cyclin D1 translocations found respectively in follicular lymphomas (FL) and mantle cell lymphomas (MCL), occur during V(D)J recombination. c-myc translocation, the typical hallmark of Burkitt lymphoma (BL), is linked to either CSR or SHM. Cyclin D1/D3, c-myc or c-maf translocations found in myeloma are clearly related to CSR [4]. Transcription of the IgH locus is under control of *cis*-regulatory elements [1, 2]. These transcriptional enhancers obviously intervene in oncogene deregulation during B-cell lymphomagenesis. During the past decade the roles and mechanism of actions of various IgH *cis*-regulatory enhancers have been clarified. At the same time various transgenic mice models have been developed in order to mimic human mature B-cell lymphomagenesis. A number of them use c-myc as a deregulated oncogene and the IgH 3’ regulatory region (3’RR) as an oncogene deregulator. Indeed, c-myc is well known to be both an important regulator of cell growth and apoptosis through its action on cell cycle progression, cell metabolism, cell differentiation, cell death receptor signaling and DNA damage recovery [3]. This review describes how IgH 3’RR physiological functions might play a critical role in c-myc oncogene deregulation during mature B-cell lymphomas and why 3’RR targeting would be an interesting promising strategy in human lymphomagenesis.

## IGH CIS-REGULATORY ELEMENTS

**Figure 1:**
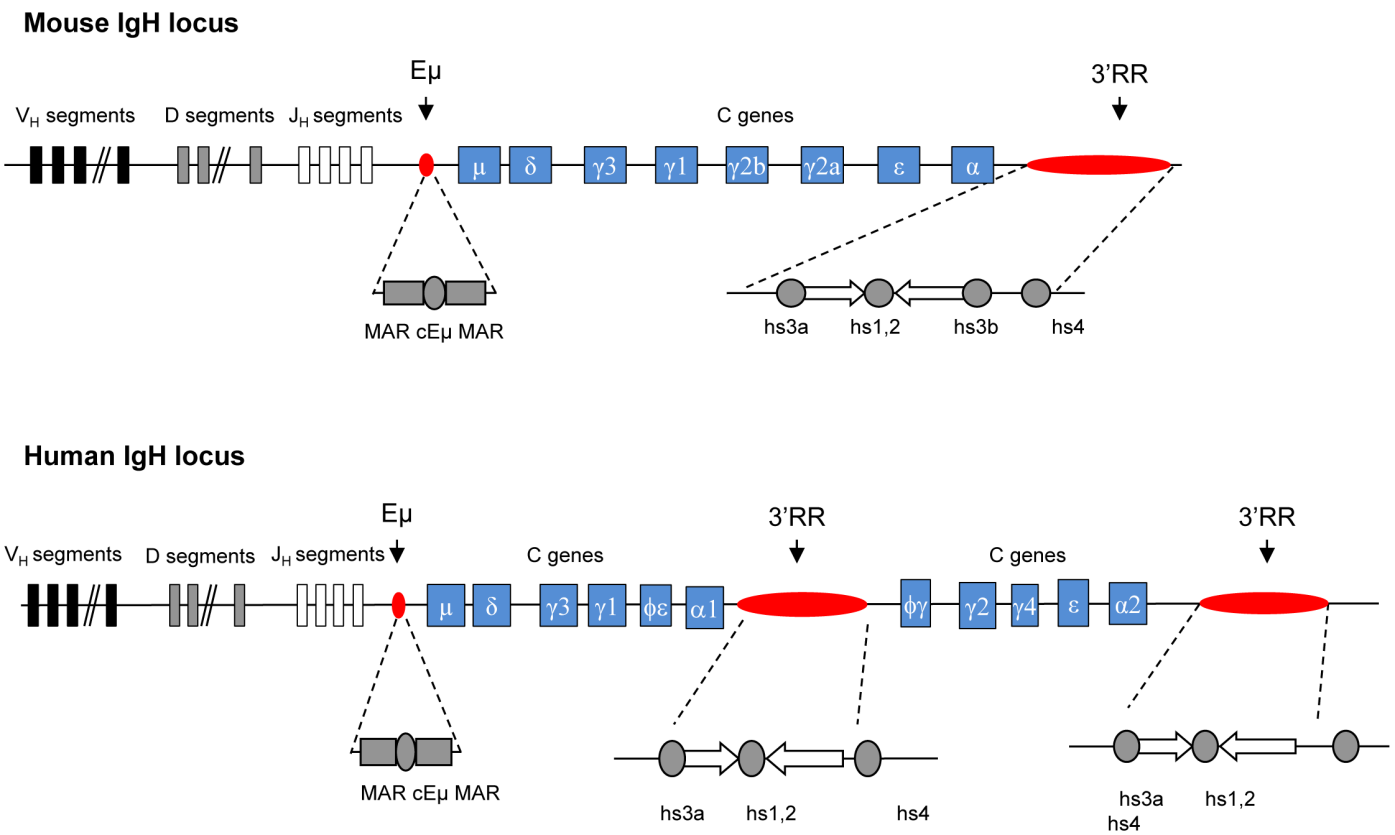
Murine and human IgH locus Upper panel: Schematic diagrams of the murine IgH locus. Locations of Eµ (with its flanking matrix attachment regions, MARs) and 3’RR (encompassing four transcriptional enhancers with flanking inverted repeats) are highlighted. Lower panel: Schematic diagrams of the human IgH locus. Locations of EµMARs and the two 3’RR (encompassing three transcriptional enhancers with flanking inverted repeats) are highlighted.

**Figure 2:**
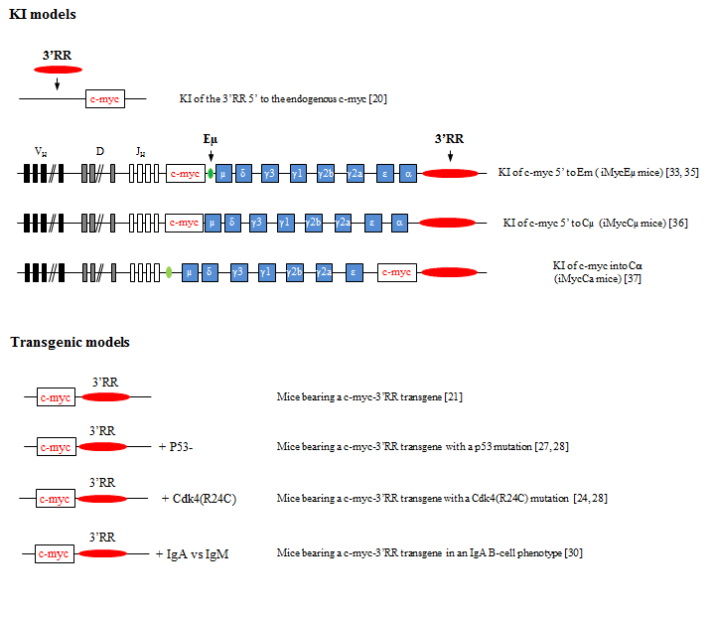
Knock-in and transgenic models of 3’RR induced c-myc lymphomogenesis A schematic representation of the various knock-in (upper panel) and transgenic models (lower panel) reporting that the 3’RR deregulates c-myc leading to B-cell lymphomogenesis. Bibliographic references are in brackets.

IgH *cis*-regulatory elements are major locus regulators (Figure 1). Two important regions have been identified. The intronic 5’ Eµ enhancer is mandatory for VDJ recombination and thus plays a key role during early steps of B-cell development [2, 5]. VDJ recombination is initiated by Rag1/Rag2 nucleases. The resulting Rag-induced DNA double-strand breaks are ideal sites for oncogene translocation. The IgH 3’RR promotes SHM [6, 7], CSR [8–10], µ transcription [11] but not VDJ recombination [12]. In germinal centers, SHM and CSR are AID-mediated modifications. SHM mainly occurs in VDJ exons for high affinity antibody generation. CSR is required for synthesis of non IgM Ig. Although these events imply different mechanisms, they both require the initiation of simple- or double-strand breaks by AID, which dramatically increase the risk of oncogene translocation. The 3’RR, as the principal AID-dependent recombination regulator, is the major player during late B-cell maturation stages. The mouse 3’RR is downstream from the IgH Cαc gene and shares a strong structural homology with 3’RR located downstream from each human IgH Cα gene (Cα1 and Cα2) [13] (Figure 2). 3’RR structural homology has been conserved during vertebrate evolution highlighting its importance during B-cell lymphopoiesis. The mouse 3’RR contains four transcriptional enhancers (hs3a, hs1,2, hs3b and hs4). hs1,2 is flanked by inverted repeat sequences and is the center of a 25-kb palindrome bound by two inverted copies of hs3 enhancers (hs3a and hs3b) [14]. hs4 is downstream from the palindrome. Each 3’RR enhancer has weak *in vitro* activity, but a synergistic and potent global effect in transgenes, especially when the palindromic 3’RR architecture is maintained [15]. Recently, sequential activation and distinct functions were reported for distal (hs4) and proximal (hs3a-hs1,2- hs3b) modules within the 3’RR [16]. Transgenic mice have shown that the 3’RR palindrome is of key importance for efficient SHM [17]. Together, the four enhancer elements are sufficient for efficient germline transcription, CSR and Ig transcription [7]. These transcriptional enhancers might thus play a role during the oncogene deregulation found in several types of mature B-cell lymphomas.

### Transgenic and knock-in models of c-myc lymphomagenesis

The *cis*-regulatory Eµ element was first suspected to be the c-myc deregulator in BL. However, Eµ-c-myc transgenic mice developed immature B-cell lymphomas [18] clearly different from a human BL which has a mature B-cell signature [19]. In human BL, c-myc translocation breakpoints are found in the VDJ (*i.e*., endemic BL with Eµ and 3’RR at the IgH locus) or in the switch µ region (*i.e*., sporadic BL with Eµ deletion and 3’RR at the IgH locus). Thus, a 3’RR was progressively suspected to be a good candidate for oncogene deregulation. Mouse and human 3’RRs share a strong structural homology [13]. Mouse models exploring the 3’RR role in B-cell malignancies should provide interesting data concerning human B-cell lymphoma development.

The first model investigating the role of IgH 3’RR in c-myc deregulation was in knock-in mouse in which 3’RR was inserted 5’ to the endogenous c-myc locus (3’RR-myc-knock-in mice) [20]. After ten months, mice developed clonal BL-like B-cell lymphomas with a B220^+^IgM^+^IgD^low/neg^ phenotype. In these mice, 3’RR stimulated endogenous c-myc transcription triggering both cell proliferation and apoptosis in pre-malignant spleen B-cells. Lymphoma development required multiple “hits” in addition to c-myc deregulation. Mutations in the p53-ARF-Mdm2 apoptotic pathways occurred during lymphoma development. Thus, only recently, has 3’RR been shown to be sufficient for c-myc deregulation leading to BL-like lymphomas.

Results with 3’RR-myc-knock-in mice were confirmed using mice having a c-myc-3’RR transgene [21]. Pre-malignant spleen B-cells from c-myc-3’RR mice proliferated more rapidly in response to mitogen signals and showed a higher apoptosis rate. At twelve weeks, c-myc-3’RR mice developed BL-like lymphomas or diffuse anaplastic plasmacytomas. BL-like lymphomas were B220^+^IgM^+^IgD^+^ with a “starry sky” appearance. Transcriptome analysis indicated alterations in the growth program and Ras-p21 pathway. Compared to 3’RR-myc-knock-in mice, the c-myc-3’RR mice developed two different phenotypes of B-cell lymphomas with two different kinetics of appearance and lacked mutations for the p53-ARF-Mdm2 apoptotic pathways [21]. In the 3’RR-myc-knock-in model, these differences imply 3’RR targeting the endogenous c-myc locus directly. The 3’RR would affect c-myc locus by destroying with its insertion some positive/negative endogenous *cis*-regulatory elements or by influencing their activation through the 3’RR long range transcriptional activity.

Interestingly, the occurrence of lymphoproliferations in c-myc-3’RR mice was sensitive to genetic background. C57Bl/6 mice developed BL-like lymphomas, while none occurred in a Balb/c background [22] known to have an allelic variant in the coding region of the p16^INK4a^ tumor suppressor gene that produces a protein with impaired activity. The regulation of cell cycle progression is managed by cyclins and cyclin dependent kinases (Cdk). Cdk4 and Cdk6 are chief catalytic subunits of the cyclin D family of proteins that govern G_1_/S phase progression. The tumor suppressor protein p16^INK4a^ and all members of the INK4 family bind specifically to Cdk4 or Cdk6 and inhibit the cyclin D-Cdk4 and cyclin D-Cdk6 complexes formation. Breeding Cdk4^R24C^ mice (a knock-in strain expressing a Cdk4 resistant to p16^INK4a^ inhibition and other Ink4 family members [23]) with c-myc-3’RR mice (that developed aggressive BL) led to the development of clonal indolent blastoid MCL-like lymphoma (CD19^+^IgM^+^CD5^+^CD43^+^CD23^-^ cells) in c-myc-3’RR/Cdk4^R24C^ mice [24]. Large amounts of Cdk4/cyclin D1 complexes were the main hallmark of these lymphomas. Although silent in non-malignant B-cells, a defect in the Ink4-Cdk4 checkpoint could contribute to lymphomagenesis in conjunction with additional alterations of cell cycle control, a situation which is reminiscent of the development of human blastoid MCL.

p53 is a transcription factor that triggers growth inhibition and apoptosis [25]. p53 inactivation is the most common feature of human tumors. The frequent loss of p53 function in human lymphomas underscores its critical role in suppressing the emergence of incipient tumors [26]. A genetic anomaly initially restricted to c-myc deregulation clearly selects tumors with a homogeneous high proliferating signature and has no need for additional alteration of apoptotic pathways. Breeding of c-myc-3’RR mice with p53-deficient mice confirmed the hypothesis that early alteration of apoptosis in a model of deregulated proliferation (*i.e*., 3’RR-driven c-myc over-expression in B-cells) induced tumor onset in a higher number of animals. Furthermore, these double mutant mice developed not only highly proliferative BL but also less proliferative types of lymphomas as observed in human patients where c-myc deregulation frequently occurs in pathologies such as MCL and plasmablastic cell lymphomas (PCL). While c-myc-3’RR mice developed non-activated CD43^-^ BL, a wider pattern of lymphomas occurred in c-myc-3’RR/p53^-^ mice including CD43^-^ BL, CD43^+^ BL, MCL-like lymphoma (IgM^+^CD5^+^CD43^+^CD23^-^ cells) and PCL (IgM^-/low^IgD^-/low^CD138^+^ cells) [27, 28]. This spectrum of tumors provided strong evidence that in the natural progression of lymphomagenesis, an initial defect in apoptosis can endow a 3’RR-mediated deregulation of c-myc to induce the occurrence of various types of mature B-cell lymphomas. This model thus accurately mimics the effect of c-myc translocation as seen in multiple human lymphomas.

Some B-cell lymphomas are associated with IgM or non IgM expression. B-cell receptor (BCR) signaling is essential for B-cell survival and response to antigens. BCR signaling varies not only among B-cell compartments but also among BCR classes. This suggests that different BCR signaling would affect lymphomagenesis. Breeding *c-*myc-3’RR mice in a genetic background (α1-knock-in mice) [29] where IgA replaced IgM (IgA inducing stronger tonic BCR signaling than IgM) led to the generation of more differentiated CD138^+^ and less proliferative B-cell lymphomas [30]. BCR class-specific signals thus affect experimental c-myc-induced lymphomagenesis. The class of the BCR often correlates with the maturity of human B-cell tumors. IgM is thus associated with less differentiated malignancies [31, 32]. IgA tumors in α1-knock-in-*c-*myc-3’RR double mutant animals indicated that a class-switched BCR signal resulted in cell transformation with a more differentiated phenotype (and the occurrence of plasma cell lymphoma), then reminiscent of human plasma cell proliferations that overwhelmingly produce class-switched Ig. This could also be pertinent to observations made in patients, where IgM^+^ malignancies are frequently more aggressive than related class-switched proliferations. Finally these results confirm the expression of the 3’RR at the plasma cell stage.

The most convincing data for the essential 3’RR role in c-myc lymphomagenesis were brought by transgenic mice with IgH-c-myc translocations [33–37]. c-myc knock-in in the mouse IgH locus just 5′ Eμ (iMycEµ mice), modeling human endemic BL, induced B-cell lymphoma development after six months. Lymphomas in iMycEµ mice had alterations in the p19^Arf^-Mdm2-p53 tumor suppressor axis, a key pathway during c-myc-induced apoptosis [33]. Authors reported complex alterations with overexpressed p19^Arf^ or lost p19^Arf^ due to biallelic deletion of the Ink4a locus associated with overexpression of Mdm2 in some tumors and sometimes elevation of a presumably dysfunctional p53. NFκB/STAT3/PI3K signaling crosstalk was later demonstrated to maintain c-myc-driven lymphomagenesis in iMycEµ mice [34]. The mouse plasmacytoma T(12;15) translocation is a well-known cancer-associated chromosomal translocation in mice that join c-myc (in chromosome 15) with the IgH locus (in chromosome 12) [38]. iMycEµ mice also mimic the T(12;15) translocation resulting in c-myc activation in murine plasmacytomas and thus also developed plasmacytomas [35]. In iMycEµ mice, c-myc is under transcriptional control of both the 3’RR and Eµ. Eµ and 3’RR have activation kinetics clearly different with Eµ acting in early B-cell development and the 3’RR in mature B-cell stages [2]. The definitive proof of an involvement of the 3’RR in B-cell lymphomagenesis was brought by iMycCµ mice carrying a c-myc knock-in in the IgH locus just 5′ of Cμ with deletion of Eµ. The 3’RR alone (without Eµ and/or putative Eµ-3’RR transcriptional crosstalk) is sufficient to deregulate c-myc expression in the B-cell lineage [36]. Plasmablastic neoplasms were rare in iMycCµ but markedly increased after breeding with transgenic Kaposi Sarcoma-associated herpes-virus IL6 (vIL6) mice [36]. In this study authors validated their hypothesis that vIL6 mice may be a driver of transformation of B-cells to malignant cells. Finally knock-in insertion of c-myc directly into Cα just 5’ to the 3’RR (iMycCα mice) produced infrequent plasma cell neoplasms (about 10%) [37]. In contrast, after breeding with transgenic mice bearing the bcl-X_L_ gene under the transcriptional Ig κ light chain 3’ enhancer, these double transgenic mice quickly developed plasma cell tumors. Bcl-X_L_ is a well-known anti-apoptotic molecule and the Ig κ light chain 3’ enhancer is of importance to drive Bcl-X_L_ expression specifically in the B-cell compartment from pre-B to mature B-cell stages. Taken altogether these knock-in models carrying c-myc at the IgH locus are prone to B-cell neoplasias of various penetrance, kinetics, and fate of knock-in bearing lymphocytes, highlighting the key role of IgH *cis*-regulatory enhancers (especially the 3’RR) for lymphoma progression. They also highlight the need for additional mutations in addition to c-myc deregulation to accelerate B-cell lymphoma development and that these “hits” target both proliferative, apoptotic and cytokine pathways.

### 3’RR knock-out models of c-myc lymphomagenesis

To elucidate 3’RR function, mice with genomic mutations were generated. Individual deletion (knock-out mice) of hs3a [39], hs1,2 [39], hs3b [40] or hs4 [41] has no effect on B-cell lymphopoiesis. In contrast deconstruction of the 3’RR palindrome structure [17] or deletion of two [17, 42], three [16] or four [8] of the 3’RR enhancers severely impaired B-cell lymphopoiesis. Mutant 3’RR mice were used in a few number of studies together with c-myc. 3’RR deleted mice showed that 3’RR was dispensable for the development of immature pro-B-cell lymphomas related to VDJ recombination-initiated c-myc translocations in double p53/Lig4 deficient mice [43]. 3’RR was not essential for the generation of IgH-c-myc translocations in double p53/Xrcc4 deficient mice. However it had an important role in c-myc transcription after IgH-c-myc translocation thus leading to mature B-cell tumors [43]. These results are in agreement with the long distance effect of 3’RR on IgH transcription at mature B-cell stages. Transcriptional enhancers of κ and λ light chain loci efficiently deregulate c-myc. Mice carrying the c-myc gene under the transcriptional control of Igλ enhancers (Igλ-myc mice) developed BL [44]. Breeding Igλ-myc mice in a 3’RR-deficient background deviated B-cell lymphomagenesis toward less mature B-cell lymphomas most probably through a 3’RR action during B-cell development [45]. Indeed during B-cell development the 3’RR is crucial for µ chain transcription and density of BCR expression at the B-cell membrane [11]. Together results from these 3’RR knock-out models indicate that deletion of 3’RR markedly reduces the development of mature B-cell lymphomas highlighting the key role of this IgH *cis*-regulatory region for lymphoma progression.

### Targeting the 3’RR: a promising therapeutic approach for c-myc induced B-cell lymphomas

The 3’RR is essential for high-rate Ig transcription [8, 11]. Therefore, it may be a key stimulator of IgH-translocated oncogene transcription. Breakpoints for oncogene translocations are sometimes several hundred kb away from the 3’RR. Data have reported that long-range interactions, through a loop structure, are implicated in both normal and abnormal regulation of 3’RR-induced gene transcription [46–48]. Of interest, physical interactions are also documented in resting and activated B-cells between Eµ and 3’RR [46]. This structure facilitates switch-switch synapsis by approaching the switch µ region (proximal to Eµ) and a downstream switch region (targeted by the 3’RR). Such loop structure is reported of importance for gene requiring a rapid transcriptional activation [46]. This suggests cooperative transcriptional effects between IgH *cis*-regulatory transcriptional enhancers that may explain why Eµ deletion between iMycEµ mice and iMycCµ mice has such an important effect on B-cell lymphoma development [33, 36]. Therefore, as previously reported by us and others [4, 43], 3’RR targeting would in theory provide a potential strategy for the treatment of some mature B-cell lymphomas. Reinforcing this hypothesis, transgenic mice carrying IgH transgenes with the 3’RR (and independently of transgene chromosomal location, length, number and orientation) have been shown to undergo myc translocation into the transgene; these translocations generating plasmacytomas [49]. Thus the IgH constant region includes all elements necessary for both myc translocation and deregulation. Data also indicate that translocation/knock-in of c-myc to another chromosome changes its nuclear localization. In human BL cells the c-myc locus is relocalized from the nuclear periphery to the central, perinucleolar position [50]. In this setting, nucleolar proteins such as nucleolin (a polypeptide of 106 KDa constituting a part of the LR1 DNA binding protein which regulates transcription and switch recombination in mammalian B-cells) [51] may play a role in c-myc activation along with IgH *cis*-regulatory elements.

Translocations in B-cell lymphomas induce epigenetic changes [52]. This observation provides the opportunity to use new classes of anti-cancer agents, epigenetic drugs (EDs), that target histone acetylation (inhibitors of histone deacetylases, HDACi) and histone methylation (EZH2 inhibitors) to treat several B-cell lymphoid malignancies, such as MCL, FL, BL and others [53]. These drugs gave promising initial results in various clinical trials [54]. For example, preclinical data support the use of HDACi in combination with other antimyeloma agents [55, 56]. Surprisingly, the scientific basis to treat B-cell lymphomas with EZH2i and HDACi is not clear. How EDs act on B-cell lymphoma remain largely speculative. Is it *via* the role of the 3’RR on oncogene transcription? Data have documented that 3’RR activation and transcription can be down-regulated by several chemicals, including isothiocyanates (known to have anti-carcinogenic properties) [57] and HDACi [58]. Studies have reported, in a model of BL translocations, that the 3’RR vastly remodelled large (up to 450 kb) domains of translocated chromatin through epigenetic mark reprogramming [59]. We recently reported that 3’RR-induced effects are largely mediated through activation of specific epigenetic marks in a 3’RR targeted DNA [10] reinforcing that targeting the IgH 3’RR would be of interest in the down-regulation of oncogene transcription. Moreover, 3’RR absence only weakly impacts chronic inflammatory ascites formation (penetrance, kinetic of development, cellular and pro-/ anti-inflammatory cytokine compositions) onto BALB/c mice in response to pristine [60]. 3’RR-deficient B-cells remain efficient to develop oil granulomas in response to pristine with no differences for granuloma numbers, cellular composition and ability to express mRNA transcripts for several pro- and anti-inflammatory cytokines [61]. Altogether these results suggest a normal role for 3’RR-deficient B-cells in the development of an acute and chronic B-cell-mediated inflammatory response to pristine. These data reinforce the hypothesis which considers 3’RR as an interesting target for anti-lymphoma drug therapy with only low adverse effects on normal inflammatory and immune responses.

## CONCLUSIONS

The 3’RR is of key importance for SHM, CSR and µ chain transcription. It follows that it is potentially of key importance as a deregulator for IgH-translocated oncogenes, even when breakpoints are hundreds of thousands bp away from the 3’RR. Long-range interactions through loop chromatin structures are common mechanisms of both normal and abnormal 3’RR-mediated gene transcription. Data have documented interactions between the 3’RR, Eµ and the IgH locus in both normal B-cells and lymphomas. Mice transgenic for IgH-c-myc translocations have highlighted the important contribution of 3’RR in the development of mature B-cell lymphomas. Data obtained with these mice are transferable to human lymphomagenesis with respect to the high structural homology between mouse and human 3’RR. Thus, 3’RR targeted inhibition may be a potential therapeutic strategy for mature B-cell lymphoma treatments. Mouse models described herein may be useful tools for both *in vitro* and *in vivo* studies of 3’RR down-regulation treatments. Future studies will be necessary to clarify the rationale for treatment of B-cell lymphomas with HDACi and EZH2i: 1) by studying the biological functions and mechanisms of action of Eµ and 3’RR and their cross-talk in the c-myc deregulation during experimental lymphomagenesis; 2) by identifying changes in the epigenome network occurring in lymphoma cells throughout the IgH locus; 3) by *in vivo* and *in vitro* testing of the role of various HDACi and EZH2i in the development of B-cell lymphomas; 4) by identifying genetic and epigenetic changes occurring in lymphoma cells upon acquisition of drug resistance during ED treatments; and 4) by identifying molecular mechanisms of these changes. Over the past decade convincing demonstrations of the 3’RR contribution to mature c-myc-induced lymphomagenesis have been made using transgenic models. They may become now useful models for the study of treatment of B-cell lymphomas.
